# Generation and characterization of HLA-A2 transgenic mice expressing the human TCR 1G4 specific for the HLA-A2 restricted NY-ESO-1_157-165_ tumor-specific peptide

**DOI:** 10.1136/jitc-2021-002544

**Published:** 2021-06-04

**Authors:** Eugene Shenderov, Matheswaran Kandasamy, Uzi Gileadi, Jili Chen, Dawn Shepherd, James Gibbs, Gennaro Prota, Jonathan D Silk, Jonathan W Yewdell, Vincenzo Cerundolo

**Affiliations:** 1 MRC Human Immunology Unit, Weatherall Institute of Molecular Medicine, Radcliffe Department of Medicine, Oxford, UK; 2 National Institute of Allergy and Infectious Diseases, National Institutes of Health, Bethesda, Maryland, USA; 3 Department of Oncology, Sidney Kimmel Comprehensive Cancer Center at Johns Hopkins University, Baltimore, MD, USA; 4 Next Generation Research, Adaptimmune, Abingdon, UK

**Keywords:** immunotherapy, adoptive, immunity, cellular, CD8-Positive T-Lymphocytes

## Abstract

**Background:**

NY-ESO-1 is a tumor-specific, highly immunogenic, human germ cell antigen of the MAGE-1 family that is a promising vaccine and cell therapy candidate in clinical trial development. The mouse genome does not encode an NY-ESO-1 homolog thereby not subjecting transgenic T-cells to thymic tolerance mechanisms that might impair in-vivo studies. We hypothesized that an NY-ESO-1 T cell receptor (TCR) transgenic mouse would provide the unique opportunity to study avidity of TCR response against NY-ESO-1 for tumor vaccine and cellular therapy development against this clinically relevant and physiological human antigen.

**Methods:**

To study in vitro and in vivo the requirements for shaping an effective T cell response against the clinically relevant NY-ESO-1, we generated a C57BL/6 HLA-A*0201 background TCR transgenic mouse encoding the 1G4 TCR specific for the human HLA-A2 restricted, NY-ESO-1_157-165_ SLLMWITQC (9C), initially identified in an NY-ESO-1 positive melanoma patient.

**Results:**

The HLA-A*0201 restricted TCR was positively selected on both CD4^+^ and CD8^+^ cells. Mouse 1G4 T cells were not activated by endogenous autoimmune targets or a large library of non-cognate viral antigens. In contrast, their activation by HLA-A2 NY-ESO-1_157-165_ complexes was evident by proliferation, CD69 upregulation, interferon-γ production, and interleukin-2 production, and could be tuned using a twofold higher affinity altered peptide ligand, NY-ESO-1_157-165V_. NY-ESO-1_157-165V_ recombinant vaccination of syngeneic mice adoptively transferred with m1G4 CD8^+^ T cells controlled tumor growth in vivo. 1G4 transgenic mice suppressed growth of syngeneic methylcholanthrene (MCA) induced HHD tumor cells expressing the full-length human NY-ESO-1 protein but not MCA HHD tumor cells lacking NY-ESO-1.

**Conclusions:**

The 1G4 TCR mouse model for the physiological human TCR against the clinically relevant antigen, NY-ESO-1, is a valuable tool with the potential to accelerate clinical development of NY-ESO-1-targeted T-cell and vaccine therapies.

## Introduction

Cancer immunotherapy has shown great promise in the clinical setting. It consists of three pillars that each have demonstrated variable benefit: checkpoint inhibitors,^1^ adoptive transfer of tumor specific T cells directly^2^ or via bone marrow transplantation, and cancer vaccines.^3^ CD8^+^ T lymphocytes play an important role in all these therapeutic approaches, as tumor CD8^+^ T cell infiltration is a hallmark of responsiveness to immunotherapy.^4^ Further, adoptive transfer of autologous tumor-infiltrating CD8^+^ lymphocytes (TIL) can mediate antitumor immunity.^2 5 6^ These cells recognize antigen via the interaction of surface T cell receptors (TCRs) with short peptides presented by MHC class I molecules (pMHC complexes).

Efficient in vivo elimination of tumor cells and virus infected cells is correlated with T cell avidity for target cells,^7 8^ due primarily to TCR affinity, with contribution from costimulatory and cellular adhesion molecules.^9 10^ Consequently, the affinity/avidity of the TCR-pMHC interaction has been ascribed a critical role in T cell antigen recognition, activation, expansion, and memory generation.^11 12^ To date, the main work on parsing the contribution to affinity/avidity antitumor immunity has been limited to in vitro^12 13^ approaches, and is largely based on OT-I (C57BL/6-Tg(TcraTcrb)1100Mjb/Crl) TCR transgenic cells, which represent a highly artificial system for studying human tumor immunity, as it is based on recognition of a mouse class I molecule binding a chicken derived peptide, an extreme example of an avian to mammalian xenoantigen.^11^

Here, we describe a new tool for scrutinizing the detailed role of affinity and avidity in shaping the quality of tumor antigen associated CD8^+^ T cell antitumor effector function and immunogenicity, in vitro and in vivo. To this end we generated and characterized a humanized TCR transgenic mouse (designated A2Eso1G4) encoding the 1G4 TCR specific for HLA-A2 complexed with the NY-ESO-1_157-165_ peptide SLLMWITQC (9C). This TCR (hereafter referred to as m1G4 TCR) was initially identified from an NY-ESO-1 (New York esophageal squamous cell carcinoma 1) positive melanoma patient by our group.^14^ NY-ESO-1 is a highly immunogenic, tumor-specific human germ cell antigen of the MAGE-1 (Melanoma-associated antigen 1) family that is a promising vaccine and cell therapy target for tumor immunotherapy.^15^ NY-ESO-1 is 180 residue protein composed of a hydrophobic C-terminus and glycine-rich N-terminus. It has no identified functional or binding domains, and its presence has been localized mainly to the cytoplasm and somewhat to the nucleus using tissue immunohistochemical stainings.^16^ In general, NY-ESO-1 is exclusively expressed in germ cells and trophoblasts, but is upregulated in 20%–70% of cancers, including 20%–80% of all melanoma, lung, esophageal, liver, gastric, prostate, ovarian, bladder, and synovial sarcoma cancers—leading it to be classified as a cancer testis (CT) antigen. Rarely, it is expressed by lymphomas, colorectal, pancreatic and renal cancers.^17–19^

Despite being the basis for 34 human clinical trials to date, including promising NY-ESO-1 T cell phase I/II trials,^20–23^ no CT, NY-ESO-1-specific, stable TCR mouse model has been described. Existing HLA-A2 TCR transgenic mouse models are specific for differentiation antigens gp100^24^ and tyrosinase^25^ rather than CT antigens, and do not have the broad array of high-affinity TCRs((12;26 or the library of high affinity altered peptide ligands (APLs)^14 27^ available for the 1G4 TCR. Additionally, the mouse genome does not encode an NY-ESO-1 homolog thereby not subjecting transgenic T-cells to thymic tolerance mechanisms that might impair in vivo studies. This allows the unique opportunity to use the 1G4 mouse model to study avidity of TCR response against NY-ESO-1 for tumor vaccine and cellular therapy development against this clinically relevant and physiological human antigen.

## Materials and methods

### Generation of NY-ESO-1 TCR transgenic mice

Transgenic mice with TIL 1G4 TCR reactive to human NY-ESO-1 were developed on a C57BL/6 background Genomic DNA was isolated from the NY-ESO-1-specific CTL line 1G4, an HLA-A2 +human NY-ESO-1_157-165_ (SLLMWITQC) specific CD8^+^ T cell line.^28^ The genomic V-J and V-D-J genomic regions of the TCR α- and β-chains were cloned, sequenced, and subcloned into the TCR cassette vectors described previously^29^ using XmaI/SacII and XhoI/SacII respectively. The vectors were then coinjected into C57BL/6 embryos yielding transgenic founder lines using the transgenic core facility at the National Institutes of Health/National Cancer Institute Laboratory Animal Sciences Program (Maryland, USA). Transgenic animals were identified by Southern blot analysis performed on tail snips. Animals were maintained in pathogen-free facilities and under the approved procedures of the Institutional Animal Care and Use Committee. DNA sequence analysis was performed utilizing VectorNTI (Invitrogen, USA). Experiments that were performed in Biomedical services in University of Oxford were carried out under Home office licence PBA43A2E4.

### αβ 1G4 Chimeric TCR Construct Transfection of BW 58 TCR α-/β-, CD4 -/-, CD8 -/- mouse thymoma cells

Transfections were carried out utilizing Amaxa (Lonza Cologne AG, Germany) with Solution V and program A-23. 2.25 µg pTα, 2.25 µg pTβ, and 0.5 µg human CD8 pIRES vectors were mixed in 100 ul Sol. V and electroporated together. Cells were grown for 2 days in RPMI 1640 (GIBCO, Invitrogen) +10% fetal bovine serum (FCS) at 5% CO_2_ and then stably selected in media supplemented with G418 1 mg/mL (Melford, Suffolk, UK). Transfected lines were frozen in freezing media (10% dimethylsulphoxide (DMSO), 90% FCS) and stored in liquid nitrogen. Samples were bulk sorted.

### Isolation of mouse genomic DNA for genotyping and transgenic HLA-A*0201 HHD monochain integration site analysis

Mouse ear punches or 0.5 cm tail snips were processed using ArchivePure DNA Mouse Tail Kits (5 Prime, Gaithersburg, Maryland, USA) according to manufacturer’s directions or using in-house lysis buffers and Proteinase K. To genotype for HLA-A*0201 homozygous mice after crossing the founder 1G4 transgenics onto the beta2 microglobulin (beta2m) HLA-A2.1 monochain transgenic H-2Db beta2m double knockout mice (HHD) line,^30^ the HLA-A*0201 monochain’s site of integration was identified utilizing the GenomeWalker Universal Kit (Clontech and^31^). PCR primers and conditions for genotyping shown in online supplemental table S1.

10.1136/jitc-2021-002544.supp1 Supplementary data

### Fluorescent in situ hybridization

Aseptically collected mouse ear biopsies were washed three times in Ca and Mg-free phosphate-buffered normal saline (PBS; pH 7.1), resuspended in 400 µL of collagenase type 1 (4000U/ml in R1 supplemented with 1.5 mL of Dulbecco’s Modified Eagle Media (GIBCO/BRL, Gaithersburg, Maryland, USA) and 100units/ml penicillin and 100 ug/mL streptomycin and 50 ng/mL gentamicin sulfate) in 15 mL Falcon tubes, and incubated for 2 hours at 37° digest tissues. Samples were briefly vortex at 1 hour, 1.5 hours, and 2 hours. After 2 hours, supernatants were collected and 1/10th volume of fetal bovine serum (FBS) was added to neutralize collagenase activity. The resulting cell suspensions was centrifuged at 2600 g for 5 min and cell pellets were resuspended in 10 mL of D0 supplemented with 100 U/mL Penicillin-and/100 U/mL streptomycin (GIBCO), 10% heat-inactivated FBS (HyClone), and 50 µg/mL Gentamicin (Cellgro, Medlatech, Virginia, USA), and cultured in NUNC 50 mL flasks (Nalge Nunc International, Rochester, New York, USA). Fluorescent in situ hybridization (FISH) was performed as previously described.^32^

### pMHC and tetramer production

A2K^b^ tetramers containing mutations at residues 226 and 227, mutant 226/227AK A2K^b^ monomer construct, were expressed in *Escherichia coli* as inclusion bodies and refolded and purified with SLLMWITQV peptide and β2m as previously described.^33^

### Generation of full-length NY-ESO-1 recombinant vaccinia virus encoding SLLMWITQC (9C), SLLMWITQV (9V), and SIINFEKL epitopes

The QuikChange II XL Site-Directed mutagenesis kit was used with a pHR-SIN vector containing the 560Kbp full-length NY-ESO-1 cDNA and primers specified to generate 9V epitope encoding full-length NY-ESO-1 using primers P1 and P2 (online supplemental table S2). Intermediate product NY-ESO-1-Ub-mCherry 9C and 9V constructs were generated using overlapping PCR in four steps as depicted in online supplemental table S2. Step 1 using mCherry as template. Step 2 using ubiquitin cDNA template. Step 3 using NY-ESO-1 products from P1 and P2. Step 4, final constructs from step 3 were fused into individual pSC11 vectors^34^ previously digested with SalI / NotI using an In-Fusion V.2.0 CF liquid PCR cloning kit (Clontech, Mountain View, California, USA). Recombinant Vaccinia Virus (Western Reserve [WR] Strain) was constructed according to established protocols.^35^ VV-Ub-SLLMWITQC, VV-Ub-SLLMWITQV, and VV-Ub-SIINFEKL were constructed to allow liberation of peptide by cytosolic ubiquitin hydrolase as previously described^36^

### Peptides

NY-ESO-1_157-165_ SLLMWITQC and SLLMWITQV, as well as OVA257-264 (SIINFEKL) peptides were synthesized by American Peptide Company (California, USA). Peptides were dissolved at 10 mg/mL in DMSO (ATCC, USA).

### Cell culture

The B5 cell line was generated by injecting methylcholanthrene (MCA) dissolved in peanut oil subcutaneously into HHD mice, letting tumors develop spontaneously, and growing out a cell line. The B5 cell line was transduced with pHR SIN lentivirus expressing NY-ESO-1 to form the 1F4 cell line by limited dilution cloning and testing for NY-ESO-1 expression. NY-ESO-1 expressing 1F4 and B5 cell lines were maintained in RPMI media (R-10) with 10% FBS and penicillin/streptomycin (100 units/mL). HHD MCA and T2 (ATCC) cells were cultured in RPMI 1640 (GIBCO, Invitrogen)+10% FCS. Cultures kept at 5% CO_2_. All reagents were obtained from Sigma-Aldrich (USA).

### Preparation of single cell suspensions of blood, spleen and lymph nodes for flow cytometry

After euthanization, blood was quickly collected from mice by cardiac puncture and subsequently subjected to RBC lysis. Then the cells were washed three times in fluorescence-activated cell sorter (FACS) buffer before stained for tetramer or surface molecules. Mice spleen and lymph nodes were carefully harvested and mashed through a 70 µm cell strainer with a plunger. After two washes in FACS buffer (PBS containing 1% FBS and 2 mM Ethylenediaminetetraacetic acid [EDTA]), the cells were subjected to RBC lysis (eBiosciences) for 10 min, followed by two washes with FACS buffer.

### Immunophenotyping experiments

After euthanization, murine spleen, mesenteric lymph nodes and inguinal lymph nodes were harvested and crushed gently on a 70 µm cell strainer using a 1 mL syringe plunger. After two washes in fluorescence-activated cell sorter (FACS) buffer (PBS containing 1% FBS and 2 mM EDTA), the cells were subjected to red blood cell (RBC) lysis (eBioSciences)) for 5–7 min, followed by two washes with FACS buffer. The single-cell preparations were resuspended in FACS buffer containing 10 µg/mL Fc receptor block and incubated for 15 min. For dendritic cell (DC), macrophages and B cell analysis, lymph node and splenocytes were stained with antibodies against the following multiple surface antigens: anti-CD11c, anti-MHC-II, F4/80, CD11b and CD19. For T cells, natural killer (NK) and natural killer T cell (NKT) cell analysis, cells were stained with anti CD3, anti CD4, anti CD8, anti NK1.1 and anti NKp46 antibodies. Dead cells were stained with Live/Dead fixable near-infrared staining kit (Life Technologies) in PBS for 30 min at 4°C. ice. Surface-stained samples were fixed with FACS buffer containing 0.1% formaldehyde before acquisition. Data analysis was performed using FlowJo software (Treestar).

### 1G4 T cells proliferation assay

Pan T cells or CD8^+^ T cells from 1G4 mice spleen were enriched using MACS beads (Pan T cell isolation kit II and CD8a isolation kit, Miltenyi Biotec) and labeled with 5 µM CellTrace Violet (CTV) (ThermoFisher Scientific) following manufacturer’s instructions. Approximately 2×10^6^ CD8^+^ T cells or 4×10^6^ pan T cells in 200 µL volume were adoptively transferred to HHD mice. After a day postadoptive transfer, mice were infected with 10^6^ PFU of recombinant vaccinia virus (WR strain) expressing wild type NY-ESO-1 (NY-ESO-1 9C) or NY-ESO-1 with the replacement of cysteine amino acid with valine at 165th position (NY-ESO-1 9V), or vaccinia virus expressing SIINFEKL peptide. Spleen were harvested on day 3 postinfection for the analysis of CD8^+^T cells or CD4^+^T cells proliferation, which was determined by cell trace violet dilution.

### Ex vivo peptide stimulation and flow cytometry

Single cell suspension from spleen prepared as described previously was stimulated with 1 µM native NY-ESO-1 (9C) (SLLMWITQC), mutant NY-ESO-1 (9V) (SLLMWITQV), or SIINFEKL peptide for 6 hours in the presence of Brefeldin A (BFA) and monensin. For tetramer staining, splenocytes (2×10^6^ cells) were pelleted down in 96 well ‘U’ bottom plate and washed once in R-10. Cells were resuspended in NY-ESO-1 9V HLA A2-K^b^ tetramer in appropriate dilution and incubated at 37°C for 30 min. After tetramer staining, cells were washed two times in FACS buffer and stained for surface antigens: APC Cy7-antimouse TCR beta, FITC-anti-human TCR Vβ13.1, PerCP Cy5.5 antimouse CD4, PE-Cy7 antimouse CD8, BV421- antimouse CD44 on ice for 30 min. Dead cells were stained with fixable aqua dead cell staining kit following manufacturer’s (Life Technologies).

For intracellular staining, cells were incubated in fixation buffer (Life Technologies) for 45 min on ice, followed by two washes in permeabilization buffer (Life Technologies). Then the cells were stained with PE-antimouse interferon γ (IFN-γ) and BV605-antimouse interleukin 2 (IL-2) antibodies in permeabilization buffer for 30 min on ice. Data analyses were performed using FlowJo software (Treestar).

### Tumor models

Syngeneic tumor cells were generated from MCA induced tumors from HHD mice. NY-ESO-1 expressing tumor cells (1F4) were generated by transduction with lentivirus that express full length NY-ESO-1. NY-ESO-1 intracellular expression was confirmed by intracellular staining for NY-ESO-1 protein with 1 µg/mL of anti-NY-ESO-1 antibody (clone E978, Thermo Fisher Scientific) for 30 min at room temperature, followed by staining with Goat antimouse IgG FITC (1:1000 dilution) for 30 min on ice. 1×10^6^ NY-ESO-1 expressing 1F4 cells or control B5 tumor cells (transduced with empty lentiviral vector) were subcutaneously injected into 1G4 mice. Tumor sizes were measured with caliper using the following equation (LxWxW)/2.

For the tumor experiments in HHD mice, 10^6^ 1F4 cells were subcutaneously injected into the right flank on day 0. On day 6, some mice received 1×10^5^ naïve CD8^+^ T cells isolated from 1G4 mice by negative selection and subsequently were infected on next day either with 10^6^ pfu of recombinant vaccinia virus (rVV) NY-ESO-1 or rVV SIINFEKL by intravenous tail administration. In order to analyze for potentially confounding VV induced antitumor response, some tumor cells injected mice were infected with 10^6^ pfu of rVV NY-ESO-1 or rVV SIINFEKL without adoptive transfer of m1G4 CD8^+^ T cells. Tumor volume was determined by caliper measurements using the following equation (LxWxW)/2. Survival end point was 1.2 cm^3^.

Confirmation of NY-ESO-1 HLA-A2 dependent presentation: B5 cells or 1F4 cells were washed in FACS buffer and preincubated with anti-HLA-A2 antibody (BB7.2) or isotype antibody in specified concentrations for 45 min before coculture with 1G4 CD8 +T cells at an E:T ratio of 3:1 ratio in Iscove’s Modified Dulbecco’s media media with 10% FBS. BFA and monensin were added in the culture (1/1000) and cells were incubated overnight, followed by intracellular staining for IFN-γ secreting CD8 +T cells.

### Statistical analysis

Data were analyzed using Prism GraphPad V.9.0 software and statistical significance was determined by unpaired Student’s t-test with correction for multiple comparisons using the Holm-Sidak method. *denotes a significance of <0.05 in Student’s t-test.

## Results

### Generating and characterizing human 1G4 TCR HLA A2 transgenic mouse, A2Eso1G4

We generated chimeric TCR constructs composed of variable domains from human 1G4 T cell clone rearranged cDNA, and constant regions and transcriptional regulatory elements from mouse sequences^29 37^ (online supplemental figure S1A–D). To demonstrate surface expression and correct assembly of the chimeric 1G4 TCR, we cloned the coding sequence of chimeric 1G4 into pTα and pTβ cassettes plasmids (Kouskoff *et al*) which we cotransfected into BW58 TCRαβ deficient mouse thymoma cells. Correct surface expression was shown by staining transfected BW58 cells with the human Vβ13.1 antibody specific for the human 1G4 variable domain (online supplemental figure S1E). In addition, specific recognition of transfected BW58 cells expressing the 1G4 chimeric TCR by NY-ESO-1_157-165_/HLA-A2 tetramers and increased levels of CD3 confirmed the surface expression of the assembled TCR complex (online supplemental figure S1F, G). We further demonstrated the proper splicing of the alpha/beta TCR constructs in transfected BW58 cells (online supplemental figure S1H).

**Figure 1:**
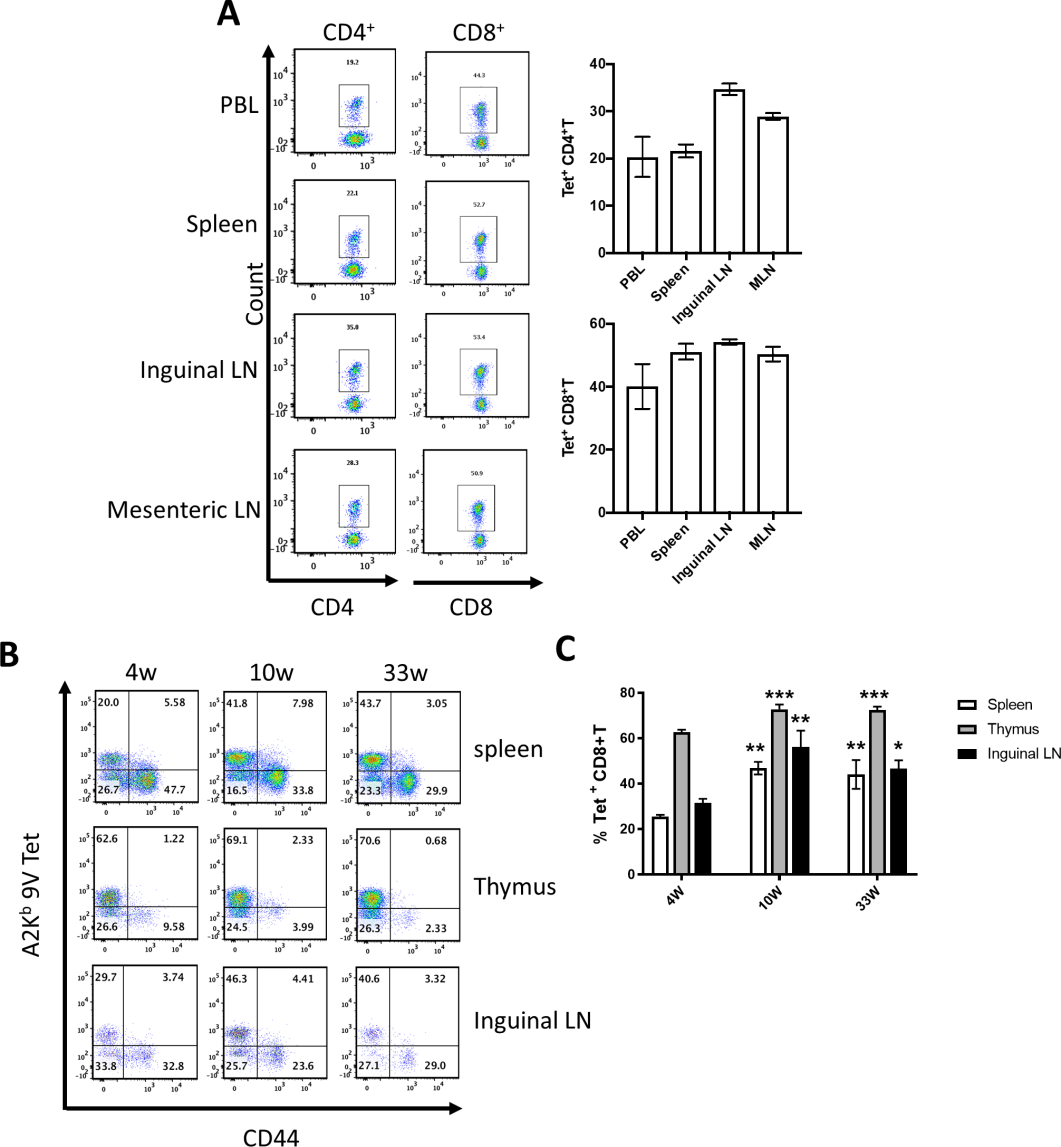
1G4 transgenic mice develop T cells specific for A2Kb/NY-ESO-1157-165. (A) NY-ESO-1157–165 specific CD4+ and CD8+ T cells from various tissues of naive 9-week-old mice were identified using Tetrameric A2Kb/NY-ESO-1157–165V. Representative FACS plots are shown for peripheral blood lymphocytes (PBL), spleens, and lymph nodes (LN) in the left panels and cumulative data from three mice per group are shown as percentage of CD4+ or CD8+ T cells on the right panels. (B, C) NY-ESO-1157–165 specific CD8+ T cells from various tissues of 4-week-old, 10-week-old, and 33-week-old naïve mice were identified using Tetrameric A2Kb/NY-ESO-1157–165V. Gating strategy for B and C: SSC-A/FSC-A>FSC-A/FSC-H single cells >LiveDead/TCRbeta >TCRbeta/CD8 >Tetramer/CD44. Representative FACS plots are shown in (B) and cumulative data of 3 mice is shown in (C). *P<0.05, **p<0.001, ***p<0.0001 against 4-week-old mice in one way ANOVA, Turkey’s multiple comparisons test. ANOVA, analysis of variance; FACS, fluorescence-activated cell sorter.

Having confirmed expression of chimeric 1G4 TCR and its ability to bind NY-ESO-1_157-165_ HLA-A2 tetramers, the 1G4 insert containing pTα and pTβ cassettes were microinjected in equimolar concentrations into C57BL/6 1-day-old embryos, and identified transgenic progeny by Southern blotting. We confirmed germ line integration of the transgenes in two distinct founders, 1G4-A and 1G4-C by FISH and PCR (data not shown and online supplemental figure S1I, J). Founder 1G4-C, with integration of the 1G4 insert into chromosome 9, was chosen for further characterization. As the 1G4 TCR is HLA-A*0201 restricted, we crossed the founder line with HLA–A*0201 expressing HHD mice, (which do not express mouse MHC class-I)^30^ to obtain mice capable of positively selecting T cells expressing this TCR. These A2Eso1G4 HHD mice were genotyped by PCR during crossing (online supplemental table S1). We confirmed by flow cytometry that mice containing the 1G4 TCRα and β transgenes on HLA-A*0201 (HHD) background were capable of positively selecting T cells, which could be stained with NY-ESO-1_157-165_/HLA-A2Kb tetramers, engineered to contain the murine CD8 binding site, in peripheral tissues (figure 1A, B).^33^ Interestingly, while most of these cells were CD8^+^ (44.3%–53.4%), a substantial population was CD4^+^CD8^-^ (19.2–35%) T cells. All NY-ESO-1_157-165_/HLA-A2 tetramer^+^ mouse T cells (m1G4 T cells) in thymus, spleen and peripheral lymph nodes displayed a naïve T cell phenotype (CD44- negative) in 4, 10 and 33-week-old mice (figure 1B, C). Detailed immunophenotyping revealed generation of all major immune cell populations though significantly decreased levels of B cells and macrophages in mesenteric lymph nodes and spleen, but not inguinal lymph nodes (online supplemental figure S2), and presence of coexpressed murine TCR without allelic exclusion given presence of Vβ13.1+cells negative for tetramer staining (online supplemental figure S3). Negligible NY-ESO-1 reactive T cells are seen by tetramer staining in the endogenous T cell pool of HHD mice (online supplemental figure S3D).

We previously demonstrated the specificity of m1G4 T cells for the NY-ESO-1_157-165_ peptide.^22^ In this study, we used a library of 5992 synthetic peptides derived from open reading frames of arenaviruses, HBV, HCV, influenza A virus, SARS virus, and vaccinia virus that are predicted to bind HLA-A*0201 with significant affinity (IC50 of <500 nM) to stimulate the m1G4 splenocytes.^26^ Incubation with target cells exposed to NY-ESO-1_157-165C_ (wild type) or its higher affinity analog NY-ESO-1_157-165V_, but not any of the other peptides tested induced CD69 expression on m1G4 splenocytes.^26^

### Immune responses of m1G4 CD8^+^ T cells to APLs of NY-ESO-1

**Figure 2:**
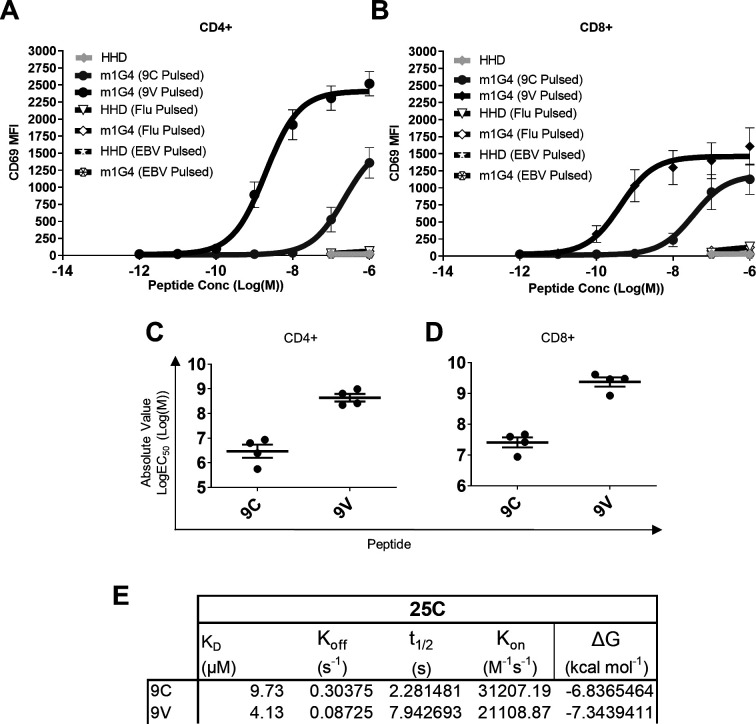
Binding properties of the m1G4 TCR to, and m1G4 CD4 +and CD8+T cell responses against, NY-ESO-1 APLs. (A, B) HHD or m1G4 splenocytes were loaded with NY-ESO-1157–165 9C (blue) or 9V (Red) peptides or irrelevant control EBV280-288 or Influenza M158-66 peptides (black). CD69 upregulation on T cells was analyzed by flow cytometry for cells stained with anti-CD4 antibody (A) or anti-CD8 antibody (B). SEM error bars on duplicate or quadruplicate assays are shown. (C, D) EC50 measurement of NY-ESO-1157–165 9C and 9V peptides interaction with m1G4 CD4+ (C) or CD8+ T cells (D) after 17 hour stimulation. Mean with SEM error bars of quadruplicate assays are shown. (E) Surface plasmon resonance affinity measurements for 1G4 TCR binding to A2 complexed with NY-ESO-1157–165 9C or 9V peptides at 25°C. APLs, altered peptide ligand; TCR, T cell receptor.

We next investigated the activation, measured by CD69 surface expression, of m1G4 CD4^+^ (figure 2A) and CD8^+^ (figure 2B) T cells using increasing concentrations of NY-ESO-1_157-165C_ peptide or its analog NY-ESO-1_157-165V_. The potency of APLs in the CD69 upregulation assay can be measured by the concentration of the peptide that gives half-maximal response (EC_50_ value in molar units (M), figure 2C, D) and confirms ~100 fold increased potency of NY-ESO-1_157-165V_ (log10 EC_50_ ~9) compared with NY-ESO-1_157-165C_ wild-type (log10 EC_50_ ~7) across both CD4^+^ and CD8^+^ T cells. In part this increased sensitivity is due to a twofold increased binding of the 1G4 TCR to the NY-ESO-1_157-165V_ APL, as revealed by surface plasmon resonance binding measurements at 25°C (figure 2C) and 37C°,^13^(4.1 vs 9.7 µM at 25°, and 7.2 vs 14.4 µM at 37°, respectively).

We then assessed whether HLA-A2 expression on human cells can activate 1G4 T cells. Culturing CD8 +T cells from m1G4 mice overnight with the TAP1/2 deficient T2 cells loaded with different concentrations of NY-ESO-1_157-165C_ wild-type peptide, revealed peptide dose dependent upregulation of CD69 and CD25 (online supplemental figure S4).

### In vivo response of A2Eso1G4 m1G4 T cells to infection-mediated antigen presentation

**Figure 3:**
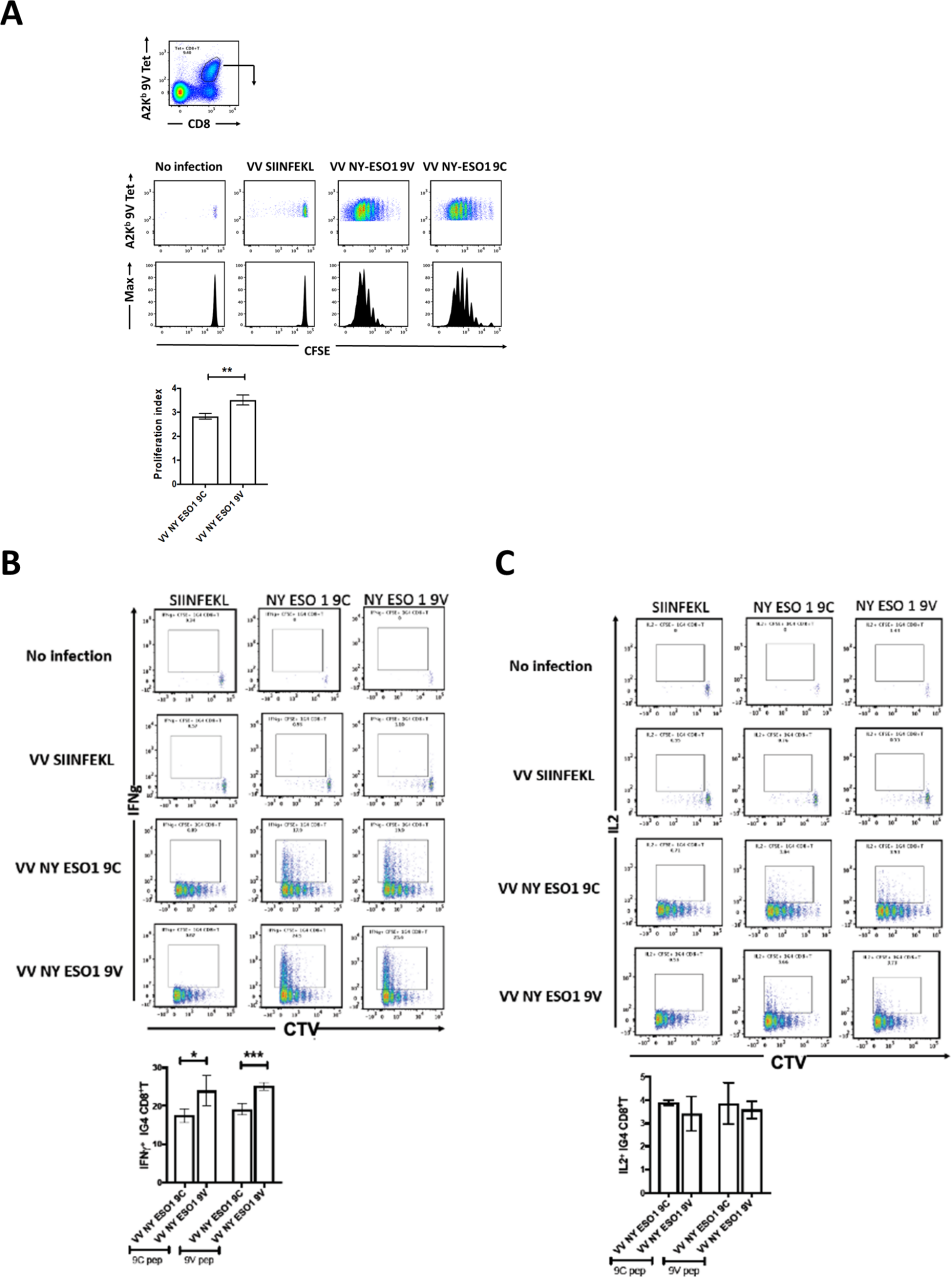
m1G4 CD8 +T cells show antigen specificity. (A) HHD mice were adoptively transferred via intravenous injection with 2×106 CellTrace Violet (CTV)-labeled naive CD8 +T cells from 1G4 mice and left as uninfected or infected intravenously with 1×106 pfu of rVV expressing full length NY-ESO-1 containing either wild type SLLMWITQC or altered peptide ligand SLLMWITQV. As control, rVV expressing SIINFEKL (from chicken ovalbumin) peptide was used. CTV dilution profiles of splenic m1G4 cells were analyzed at day three postinfection having been stained with A2Kb/NY-ESO-1157–165V tetramer. (B) Splenocytes from the infected mice were restimulated ex vivo with SIINFEKL, SLLMWITQC or SLLMWITQV peptides and IFN-γ expression or (C) IL-2 expression were analyzed by intracellular cytokine staining (ICS) and CTV dilution on day 3. For each panel, representative FACS plots are shown in the top panel and cumulative data for mouse groups (n=5) are plotted below. *P<0.05, **p<0.01, ***p<0.001 in Student’s t-test with GraphPad software. FACS, fluorescence-activated cell sorter; IFN-γ, interferon-γ; IL-2, interleukin 2.

We further investigated m1G4 T cell responses in vivo using by generating recombinant vaccinia viruses (WR) expressing full-length NY-ESO-1 with either wild-type cysteine at position 165 or valine (hereafter referred to as rVV NY-ESO-1 wild type or C165V). The constructs were designed as ubiquitin fusions (Fluorescent protein-Ub-NY-ESO-1 variant) to allow more efficient APL presentation through enhanced proteasomal degradation as previously described.^38 39^ m1G4 CD8^+^ T cells were labeled with CTV (a violet version of carboxyfluorescein succinimidyl ester [CFSE]) and transferred to HHD mice that were infected the next day with rVV NY-ESO-1 wild type, C165V, irrelevant SIINFEKL (chicken ovalbumin) peptide, or left uninfected. Three days later the splenic A2K^b^/NY-ESO-1_157-165_ tetramer positive CD8^+^ m1G4 T cell proliferation fraction was assessed and showed a statistically significant increase in the CTV proliferation index for higher affinity C165V versus wild-type vaccinia infection, and no irrelevant SIINFEKL induced proliferation (figure 3A).

Splenocytes from these mice were then re-stimulated ex vivo with the wild-type NY-ESO-1_157-165C_ peptide (9C), the higher affinity analog NY-ESO-1_157-165V_ peptide (9V) or irrelevant SIINFEKL peptide for 6 hours in the presence of brefeldin-A plus monensin and production of IFN-γ (figure 3B) or IL-2 (figure 3C) was measured. Splenocytes from mock infected or rVV SIINFEKL did not produce any IFN-γ or IL-2, while in mice infected with rVV NY-ESO-1 9C peptide an average of 17% of the m1G4 T cells produced IFN-γ and in mice infected with NY-ESO-1 9V peptide the number of IFN-γ producing m1G4 T cells was statistically higher at an average of 24%. Interestingly, the order of infection and restimulation matters with rVV NY-ESO-1 9V peptide infection producing increased IFN-γ on re-stimulation with either the wild type NY-ESO-1 9C peptide or higher affinity NY-ESO-1 9V peptide. Therefore, it seems that priming the adoptively transferred m1G4 using a higher affinity epitope (rVV NY-ESO-1 C165V) produces T cells that are qualitatively superior (producing more IFN-γ). Note that while we cannot completely exclude the possibility that some of the NY-ESO-1 A2-9V tetramer positive T cells are not adoptively transferred m1G4 but rather derived from endogenous precursor T cells, such cells are highly unlikely to have developed 3 days following priming. The percentage of IL-2 producing m1G4 T cells was much lower than IFN-γ producing, and did not differ between the mice infected with the two different versions of NY-ESO-1 expressing recombinant vaccinia virus.

**Figure 4:**
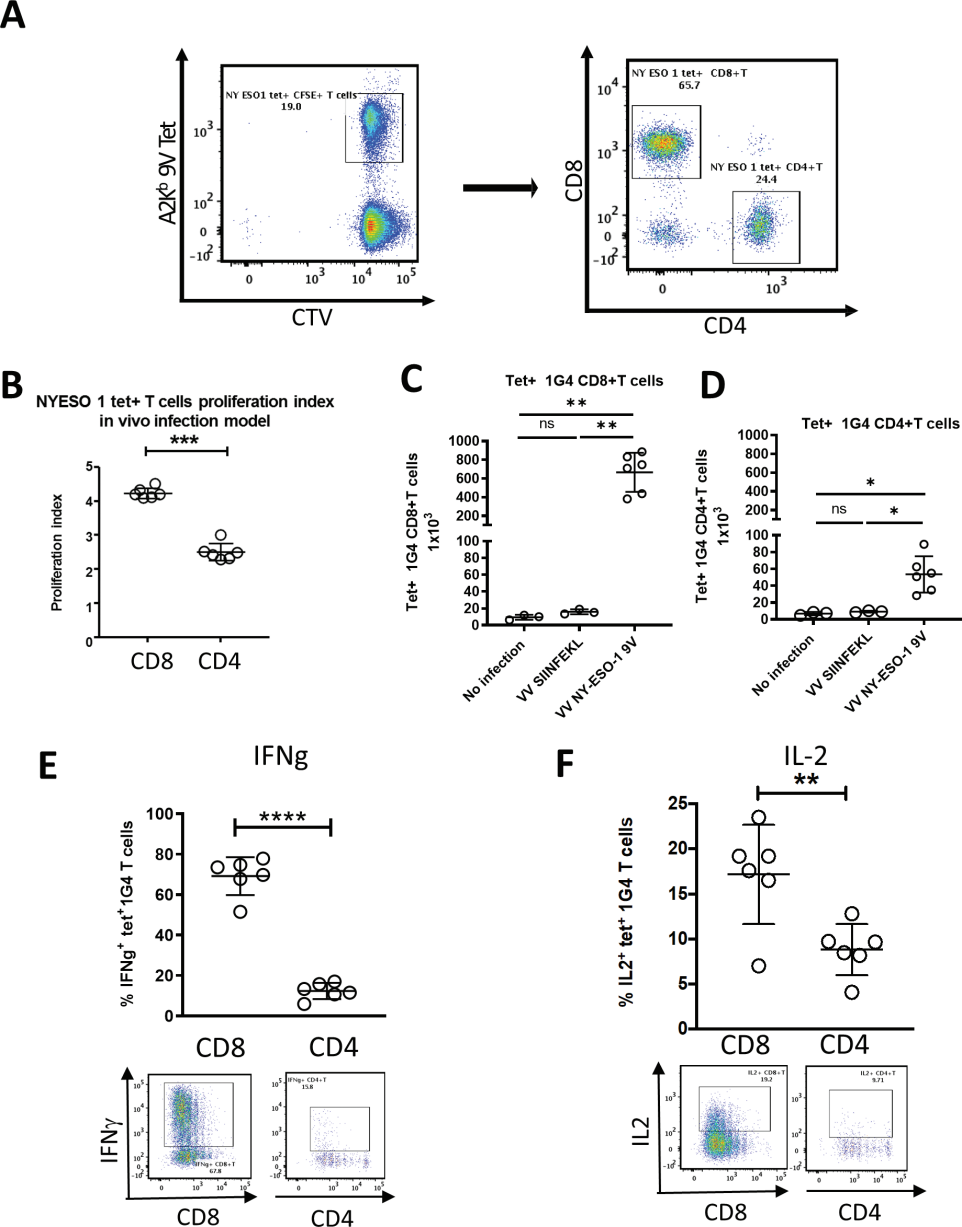
CD4+ and CD8+ T cells from 1G4 mice demonstrate in vivo antigen-specific functionality. m1G4 T cells were isolated by negative selection using Pan T cell isolation Kit (Miltenyi), labeled with CellTrace Violet (CTV) and adoptively transferred into HHD mice. Uninfected mice and mice infected with rVV NY-ESO-1 9V or irrelevant rVV SIINFEKL were then analyzed on day three post infection. (A) Splenic naïve m1G4 T cells before adoptive transfer. (B) CTV labeled splenic 1G4 CD8+ and CD4+ T cell in vivo proliferation index. Absolute number of 1G4 CD8+ (C) and CD4+T cells (D). IFN-γ (E) and IL2 (F) production after ex vivo NY-ESO-1157–165V peptide stimulation. *P<0.05, **p<0.01, ***p<0.001, ****p<0.0001 in Student’s t-test or non-parametric Kruskal-Wallis test with Dunn’s multiple comparisons test with GraphPad software. IFN-γ, interferon-γ; IL2, interleukin 2; ns, not significant.

We next compared the rVV-induced activation of CD8 +vs CD4 +m1 G4 cells labeled with cell trace violet adoptively transferred into HHD mice (figure 4A). One day after m1G4 transfer, mice were mock infected or infected with either rVV NY-ESO-1 C165V or rVV SIINFEKL. Three days post infection CD8^+^ T cells demonstrated increased proliferation in mice infected with rVV NY-ESO-1 C165V compared with CD4+ T cells, while no proliferation was noted in uninfected mice or irrelevantly infected mice with rVV SIINFEKL (figure 4B–D and online supplemental figure S5). Ex vivo splenocyte restimulation with NY-ESO-1_157-165V_ peptide (9V) for 6 hours in the presence of brefeldin-A and monensin demonstrated marked A2-9V tetramer positive m1G4 CD8 +T cell production of IFN-γ (67.8%) and IL-2 (19.2%), but substantially lower production from CD4 +T cells (IFN-γ of 15.8% and IL-2 of 9.71%) and no stimulation with irrelevant rVV SIINFEKL (figure 4E, F and online supplemental figure S5).

**Figure 5:**
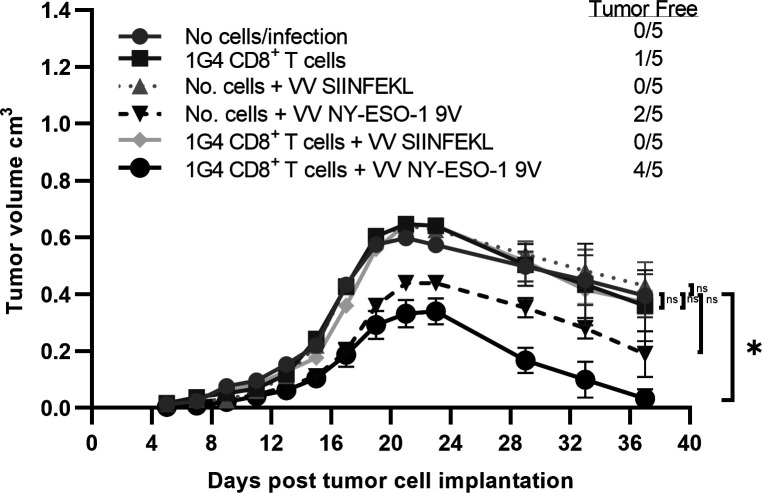
CD8 +T cells from 1G4 mice control tumor growth in vivo in an antigen specific manner following NY-ESO-1 vaccination. HHD mice (n=5 per group) were subcutaneously injected in the right flank with 1×106 full length NY-ESO-1 cDNA transduced syngeneic tumor cells (1F4 cells) on day 0. On day 6, some mice received 1×105 naïve CD8 +T cells isolated from 1G4 mice by negative selection and subsequently were infected on next day either with 1×106 pfu of rVV NY-ESO-1 9V or rVV SIINFEKL by intravenous administration. In order to analyze for potentially confounding VV induced antitumor response, some tumor cells injected mice were infected with 1×106 pfu of rVV NY-ESO-1 or rVV SIINFEKL without adoptive transfer of m1G4 CD8 +T cells. Error bars represent SEM Tumor growth curves were assessed by two-way analysis of variance (ANOVA) using the Dunnett method for multiple comparison adjustment. *P<0.05 with GraphPad software. ns, not significant.

### HHD mice demonstrate NY-ESO-1 specific control of tumor growth in vivo following NY-ESO-1 vaccination

To address the critical issue of whether m1G4 T cells, rather than endogenous CD8 +and CD4+T cells, respond to NY-ESO-1 and can suppress growth of syngeneic tumor cells expressing the full-length human NY-ESO-1 protein, we subcutaneously injected HHD mice with 10^6^ syngeneic tumor cells (MCA induced tumors from HHD mice) expressing NY-ESO-1 (1F4, figure 5). On day 6, some mice received a limited number of 1×10^5^ naïve CD8^+^ T cells isolated from 1G4 mice by negative selection and subsequently were infected on next day either with no vaccinia, 1×10^6^ pfu of rVV NY-ESO-1 9V or rVV SIINFEKL by intravenous administration to model an NY-ESO-1 directed tumor vaccine. In order to analyze for potentially confounding endogenous T cell response or VV expressed immunogenic antigen directed response, some mice received no m1G4 T cells or tumor cells injected mice were infected with 10^6^ pfu of rVV NY-ESO-1 or rVV SIINFEKL without adoptive transfer of m1G4 CD8^+^ T cells. Notably, only the HHD mice receiving both m1G4 CD8 +T cells and rVV NY-ESO-1 9V vaccination showed significant tumor clearance with 4/5 mice tumor free on day 37 (p=0.02), while none of the other tumor groups were statistically different from no cells/no infection—indicating that any variation seen was likely due to inherent immunogenicity of the NY-ESO-1-expressing MCA tumor (1F4), which primed the mouse’s endogenous T-cell repertoire against the foreign NY-ESO-1 antigen.

Direct injection of A2Eso1G4 mice subcutaneously with 10^6^ syngeneic tumor cells of either the parental line (B5) or the human NY-ESO-1 expressing derivative line (1F4) (online supplemental figure S6A) also demonstrated HLA-A2 restricted recognition of NY-ESO-1 (online supplemental figure S6B) with minimal tumor growth in the NY-ESO-1 expressing 1F4 line over 5 weeks compared with the parental B5 line, which grew uncontrolled (online supplemental figure S6C).

## Discussion

The use of vaccines to stimulate effective antitumor immune responses in humans has proven to be challenging.^40 41^ NY-ESO-1 is one of the most attractive broad tumor associated antigen (TAA) targets for human cancer vaccine development, but clinically relevant responses remain elusive.^15 42^ NY-ESO-1 targeted TCR gene therapy has been more successful with a trial by Robbins *et al* showing objective clinical responses in of patients with synovial cell sarcoma and 5 of 11 patients with metastatic melanoma.^43^ To accelerate improvements in NY-ESO-1 vaccination and cellular T-cell therapies we developed a novel TCR transgenic mouse, A2Eso1G4, using an HLA-A2 restricted TCR specific to human NY-ESO-1_157-165_ SLLMWITQC (9C) initially identified from an NY-ESO-1 positive melanoma patient’s class-I restricted CD8^+^ TIL.^14^

The m1G4 TCR was found to be expressed in CD4^+^ and CD8^+^ T cells in peripheral blood and lymphoid organs of A2Eso1G4 mice on the RAG^+^ C57BL/6 HHD genetic background. Immunophenotyping revealed intact presence of all major immune cell populations though differences were observed in numbers of B-cells, macrophages, CD8^+^ T cells, and CD4^+^ T cells in lymph node or splenic locations between HHD and 1G4 mice, with differences to be investigated further in RAG competent and knockout settings. Decreased B-cell numbers have been previously observed in mice expressing transgenic TCRs on a RAG competent background, including using the specific cassette vectors used herein, possibly due to apoptosis induced by TCR expression, accumulation, and endoplasmic reticulum stress response.^44^ Approximately, 20%–35% of CD4^+^ and 44%–53% of CD8^+^ T cells in A2Eso1G4 mice express the transgenic TCR and nearly all display the expected naïve phenotype (CD44 negative). This was true even in 33-week-old mice, pointing to the lack of self-antigen expression during this period. Accordingly, no spontaneous autoimmune manifestations were noted, and mice exhibited normal growth and aging. This is notable as a recently described HLA-A2^+^/human tyrosinase_368-376_ class I restricted, YMDGTMSQV, high affinity TCR mouse demonstrated spontaneous hair depigmentation and age progressive visual defects due to non-tolerant cross reactivity with the endogenous mouse tyrosinase peptide FMDGTMSQV.^25^ Other T cells, that are tetramer negative, exist in this RAG competent mice, some of which are CD44 positive cells and consistent with known memory-like T cells arising from presumed physiological homeostatic proliferation.^45^ The m1G4 T cells were functional, showing proliferation and cytokine secretion after activation with cognate peptide pulsed human T2 cells or mouse syngeneic splenocytes.

A2Eso1G4 m1G4 T cells exhibited NY-ESO-1 specific activation as determined by proliferation, CD69 upregulation, and IL-2 and IFN-γ production. As previously seen in transgenic TCR mice on a RAG competent background we also found absence of allelic exclusion with expression of endogenous TCR and CD4^+^ T cells expressing the m1G4 TCR,^25 37^ and demonstrated that CD4^+^ m1G4 cells can be activated through the TCR as evidenced by CD69 upregulation. However, the resultant cell is less fit compared with a CD8^+^ m1G4 cell in terms of IFN-γ and IL2 production, demonstrating a role for the CD8 coreceptor in m1G4 activation. Back-crossing of the A2Eso1G4 mice onto a RAG-/-background will be necessary to assess the efficiency of positive selection of the 1G4 TCR.

Interestingly, our adoptive transfer experiments indicate that antigen specific CD4^+^ T cell presence may improve the response of CD8^+^ T cells as evidence by improved cytokine production when m1G4 CD4^+^ T cells were cotransferred with m1G4 CD8^+^ T cells in our vaccinia infection model (comparison of CD8 functional status in figures 3 and 4). This is consistent with prior data showing transfer of LCMV-specific CD4^+^ T cells to mice with CD8^+^ T cell exhaustion from chronic infection improved CD8^+^ T cell proliferation and function,^46 47^ and is the focus of an ongoing phase I trial (NCT03691376) evaluating whether NY-ESO-1 directed CD8 TCR responses can benefit from concomitant CD4 TCR mediated help in clearance of chemo resistant ovarian cancer. The A2Eso1G4 mouse model thereby provides the opportunity to study the role of CD4 help in CD8 antitumor responses and TCR signaling in CD4 and CD8 T cells on activation with the same antigen.

Adoptive transfer of m1G4 CD8^+^ T cells into HHD mice followed by NY-ESO-1 vaccination demonstrated significant tumor clearance despite transfer of suboptimal cell doses (1×10^5^ cells), and future work can build on this by modeling various amounts of CD4 T cell help and varying the NY-ESO-1-TCR affinity dynamics using our described altered peptide libraries.^27^

A limitation of the m1G4 mouse model presented herein is the current RAG competent background that limits assessment of the efficiency of positive selection of the 1G4 TCR. We intentionally adoptively transferred suboptimal quantities of m1G4 CD8 +T cells to model a cancer vaccine system, and future experiments are required to properly modulate the balance between adoptively transferred T cell quantities, tumor size, and vaccination dose. Finally, to explore the unique properties of this mouse model a range of tumor lines will be required that vary in aggressiveness of growth, and levels of antigen expression and presentation.

In summary, we believe the novel mouse model reported in this study presents a useful TCR transgenic mouse model of tumor reactive T cells that target a clinically relevant mammalian TAA. Absence of murine NY-ESO-1 expression, prevents thymic selection against the TCR, opens the door to future higher affinity TCR mouse variants, and allows our model to serve as a viral or TAA platform to investigate basic T cell antiviral or antitumor responses, and design future tumor immunotherapy protocols, focused on T cell affinity/avidity for tumor vaccine and cellular T-cell therapy development.

## Data Availability

Data are available on reasonable request.
