# Supplementary material for: Generation and characterization of HLA-A2 transgenic mice expressing the human TCR 1G4 specific for the HLA-A2 restricted NY-ESO-1157-165 tumor-specific peptide
Source: J Immunother Cancer. 2021 Jun 4;9(6):e002544. doi: 10.1136/jitc-2021-002544 (PMC8183295; doi:10.1136/jitc-2021-002544)
Supplement: Supplementary data [file jitc-2021-002544supp001.pdf]

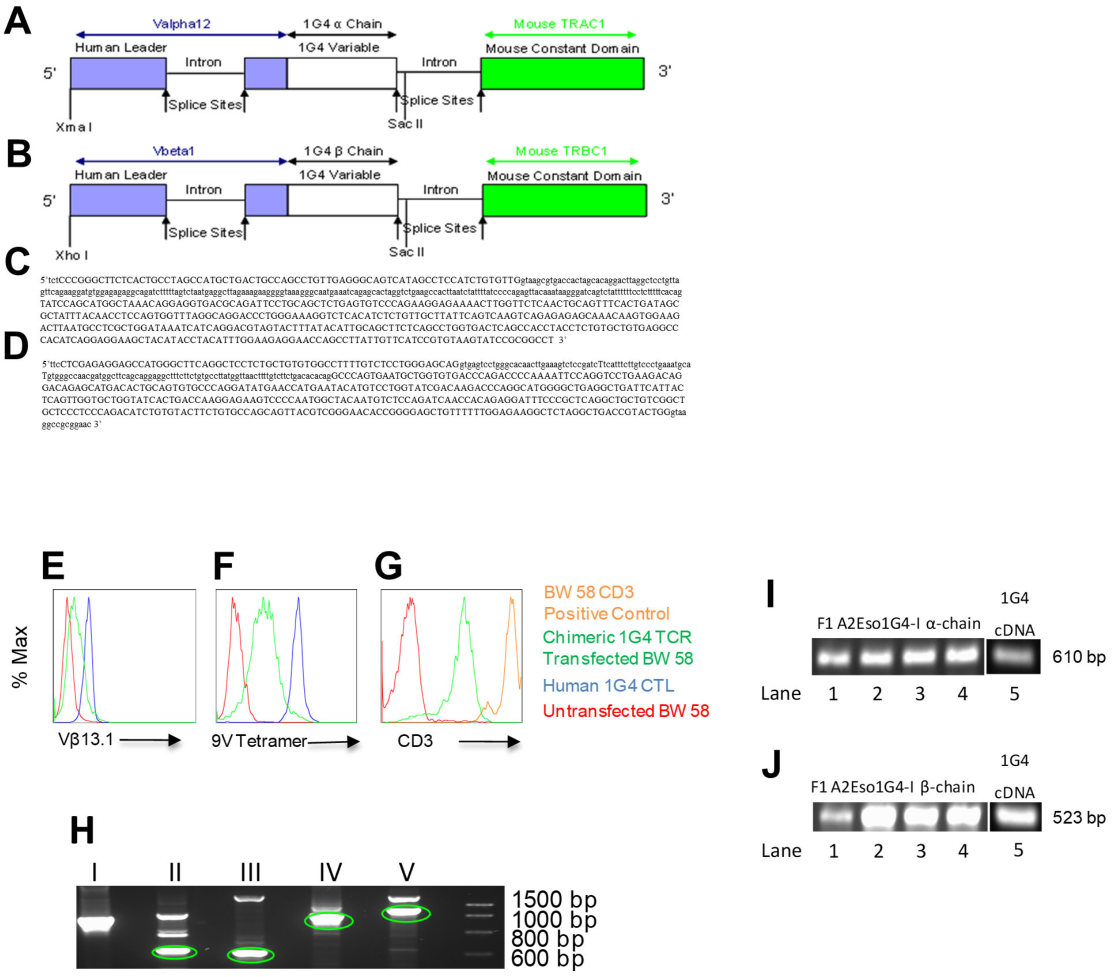

Fig. S1. 1G4 Chimeric Human/Mouse 1G4 TCR Transgenic Design, Surface Expression and Correct Splicing. **(A)** Representation of transgenic full-length, chimeric human/mouse 1G4α gene construct. **(B)** Representation of transgenic full-length, chimeric human/mouse 1G4β gene construct. **(C and D)** DNA sequences encoding 1G4 pTα and pTβ LVDJ segments respectively; introns shown in lowercase. **(E)** Comparative flow cytometry staining of the chimeric 1G4 TCR expressed in BW58 and the fully human 1G4 TCR expressed in the native human 1G4 CTL using the Vβ13.1 antibody specific for the human 1G4 variable β-chain. **(F)** Staining with the NY-ESO HLA-A2 9V tetramer. **(G)** Anti-CD3 staining. **(H)** Splicing of the alpha/beta TCR constructs was assessed in transfected BW58 cells using PCR and resolved by 1.2% agarose gel. (I) β2 actin house keeping gene positive control. (II) α TCR leader sequence splice check. (III) β TCR leader sequence splice check. (IV) Full-length α TCR. (V) Full-length β TCR. Correct bands confirmed by sequencing and encircled in green. (**I and J**) 1G4 TCR α- and β-chain germline transmission to F1 1G4 mice. Founder (F0) mice were backcrossed onto an HHD background and genotyped to confirm germline transmission. BW 58 1G4 TCR transfectants provided positive control cDNA. **(I)** TCR α-chain transgene product and **(J)** TCR β-chain transgene product from mouse genomic DNA. DNA quality confirmed by PCR analysis of mouse β2 actin for all samples.

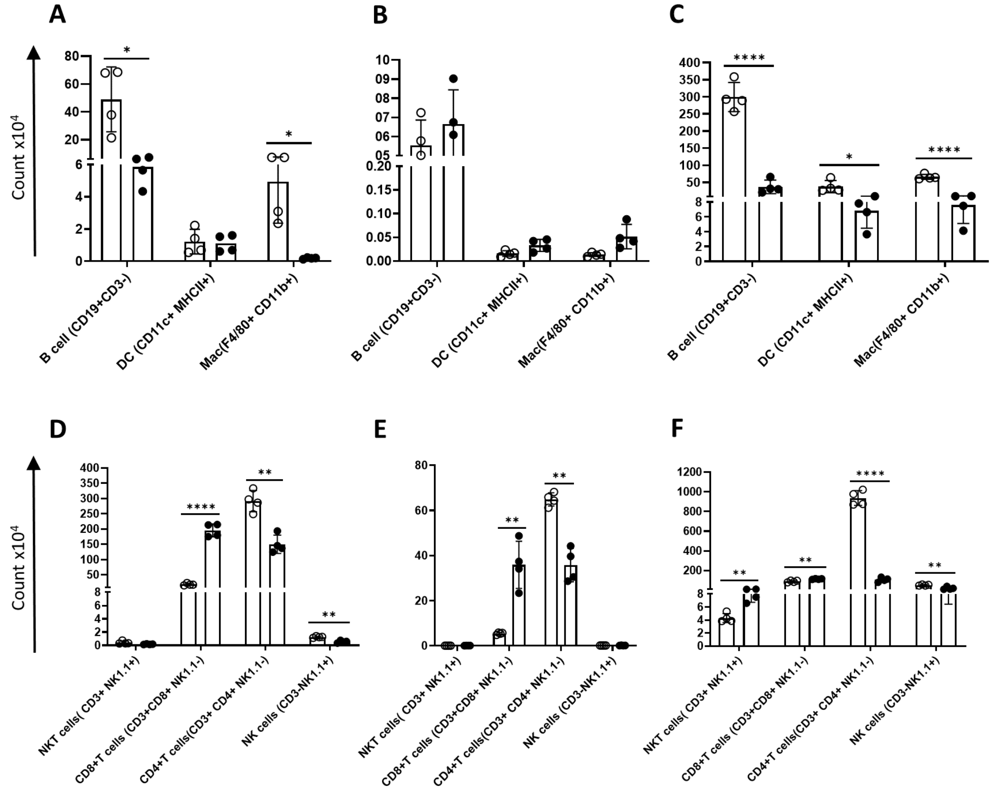

Fig. S2. Immunophenotyping of 1G4 mice reveals generation of all major immune populations. (A-C) B-cells, DCs, and Macrophages in **(A)** mesenteric lymph node, **(B)** inguinal lymph node, and **(C)** spleen. **(D-F)** NKT, CD8+ T cells, CD4+ T cells, and NK cells in **(D)** mesenteric lymph node, **(E)** inguinal lymph node, **(F)** spleen. All graphs show the mean ± SEM from n = 4 mice per group. * denote a significance of P value <0.05, ** denote a significance of P value <0.01, ***denote a significance of P value < 0.001, and ****denote a significance of P value <0.0001 in Student *t* test in GraphPad software. HHD mice (open circles) and 1G4 mice(closed circles)

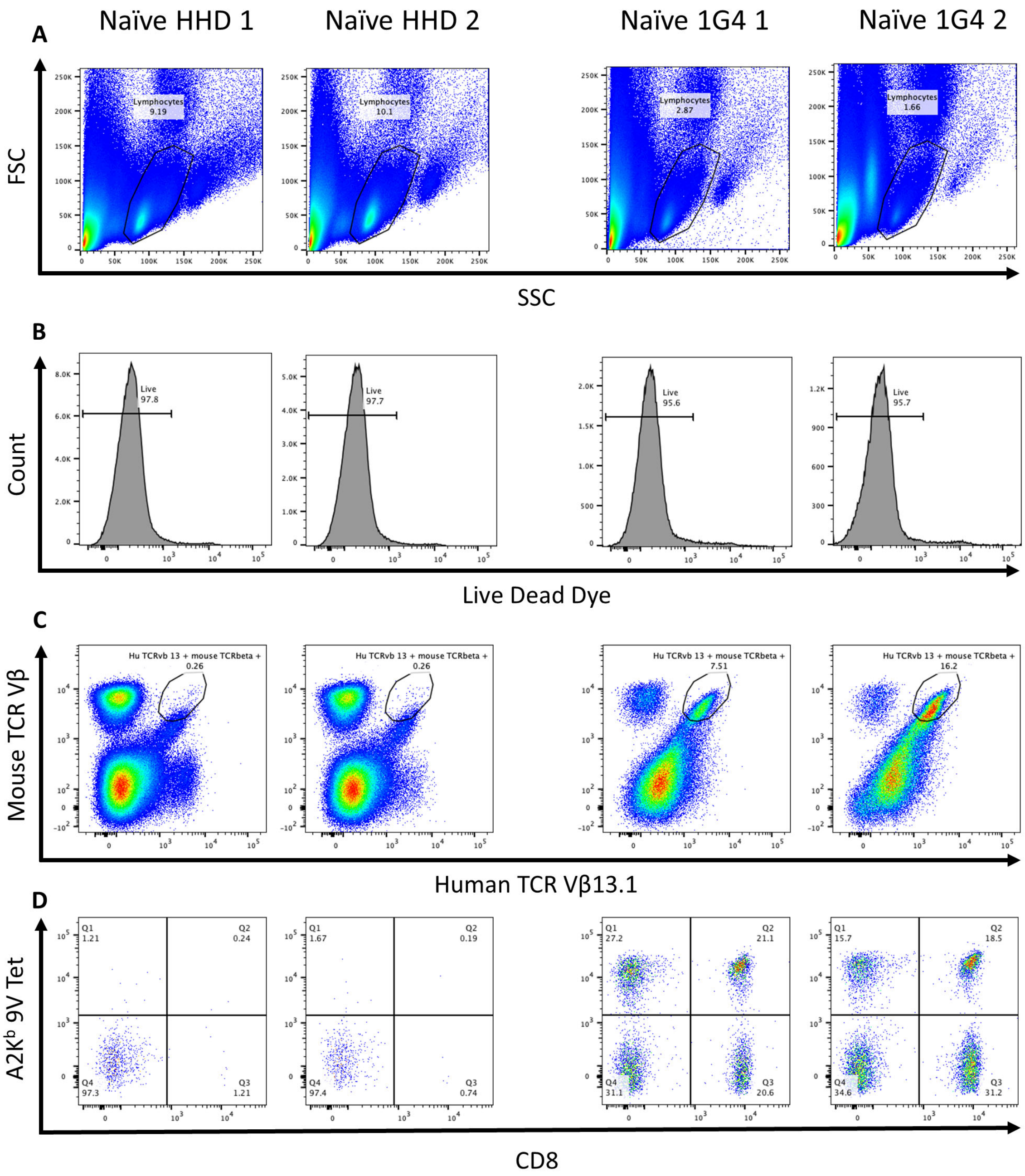

Fig. S3. 1G4 mice demonstrate specific T cells without allelic exclusion. (A) Peripheral blood lymphocyte gating using forward scatter (FSC) and side scatter (SSC), gate A depicted. **(B)** Peripheral blood live cell gating on gate A, gate B depicted. **(C)** Identification of mouse TCR Vβ and human TCR subunit specific human Vβ13.1 cells in naive HHD (n=2) and 1G4 (n=2) mouse peripheral blood lymphocytes in live cell gate B, gate C depicted. **(D)** Identification of A2Kb 9V tetramer positive and CD8 positive cells in naive HHD (n=2) and 1G4 (n=2) mouse peripheral blood lymphocytes in gate C.

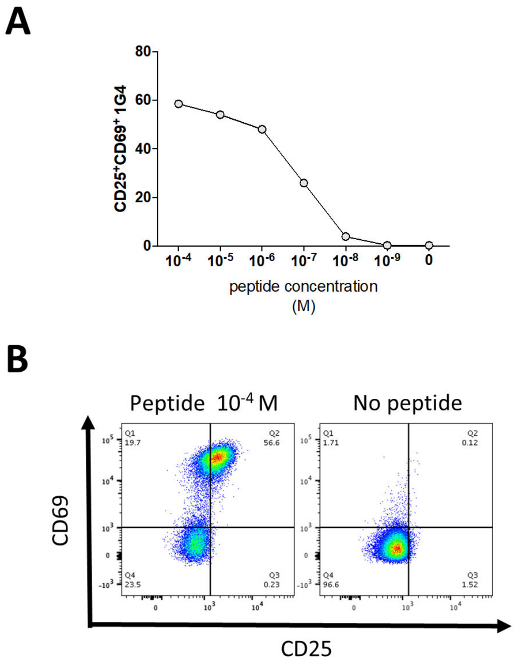

Fig. S4 NY-ESO-1$_{157\text{-}165}$ specific CD8^{+} T cell from the 1G4 mice can be activated by human cells presenting NY-ESO-1$_{157\text{-}165C}$ peptide ***in vitro.*** CD8^{+} T cells isolated from 1G4 mice were co-cultured overnight with the TAP1/2 deficient cells, T2, that were loaded with different concentration of NY-ESO-1$_{157\text{-}165C}$ peptide. **(A)** CD69 and CD25 up-regulation was analyzed by flow cytometry for cells stained with an anti-CD8 antibody, **(B)** A representative dot plot is shown (representative data is shown from two experiments).

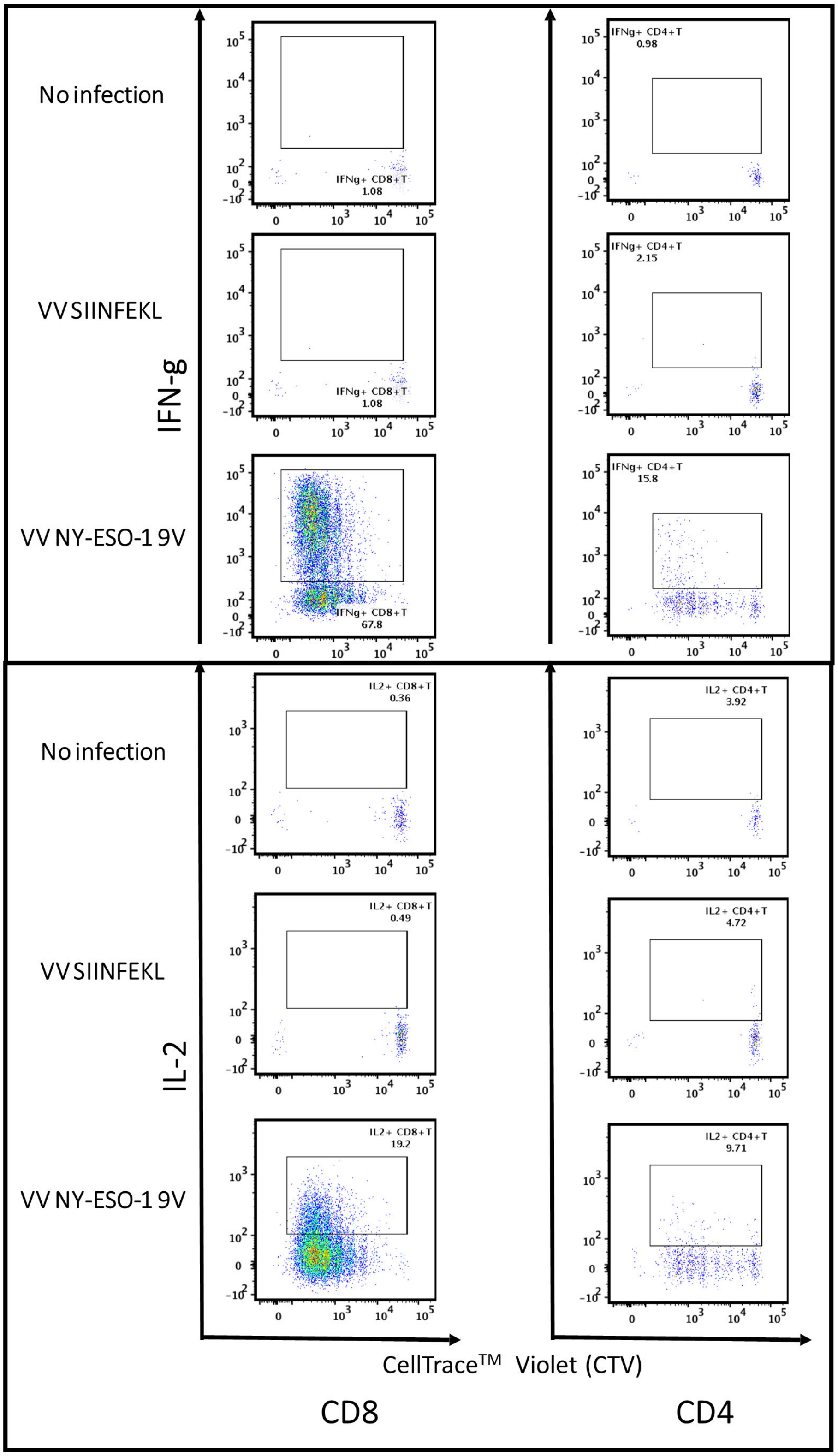

Fig. S5. CD4⁺ and CD8⁺ T cells from 1G4 mice, ex-vivo antigen specific IL-2 and IFN-γ production (supplementary data for Figure 4). IFN-g (top six panels) and IL-2 (bottom six panels) production after ex vivo NY-ESO-$1_{157\text{-}165V}$ peptide stimulation. m1G4 T cells were isolated with Pan T cell isolation kit II, labeled with CellTrace^{TM} Violet, and adoptively transferred into HHD mice. Splenocytes from uninfected mice and mice infected with rVV NY-ESO-1 9V or irrelevant rVV SIINFEKL were then stimulated with NY-ESO-$1_{157\text{-}165V}$ peptide using ICS. Representative FACS plots are shown to demonstrate the lack of stimulation in the mice that did not receive rVV NY-ESO-1 9V.

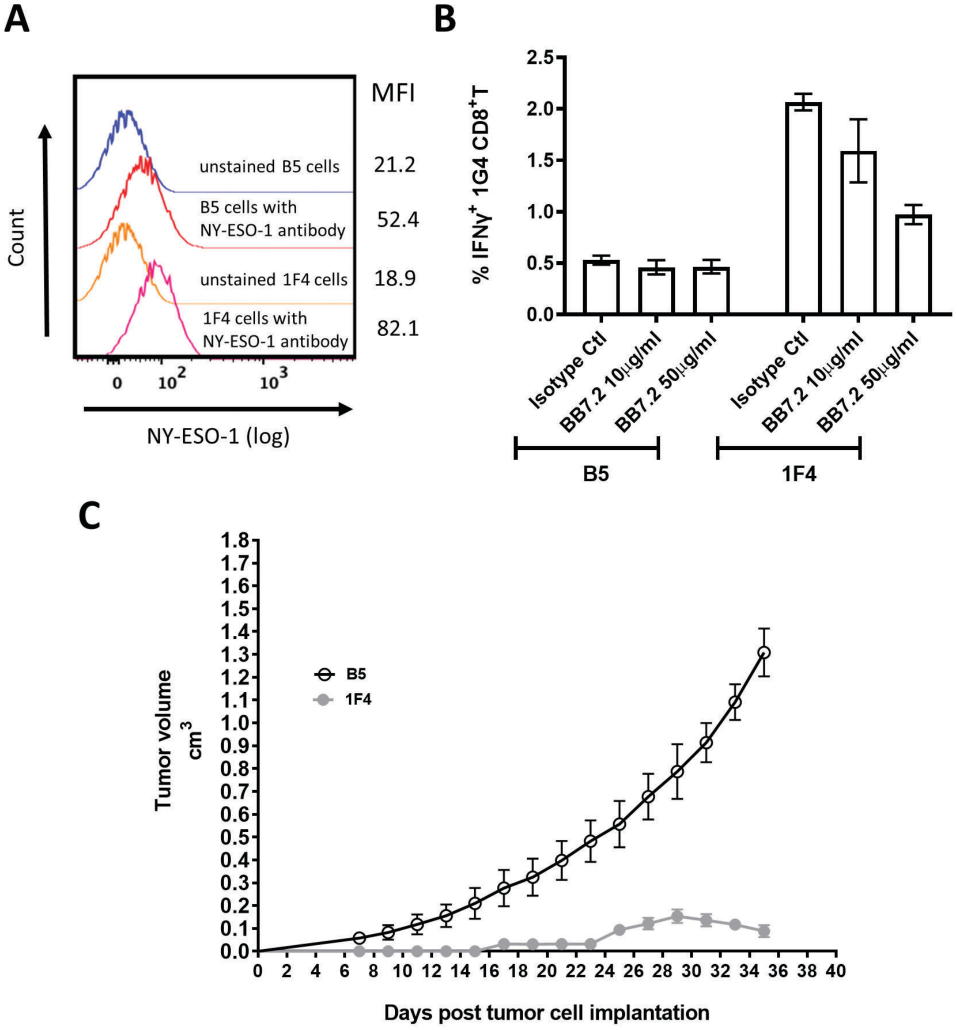

Fig. S6. T cells from 1G4 mice control tumor growth *in vivo* in an antigen specific manner with restriction by HLA-A2 expression. (A) Representative B5 and 1F4 NY-ESO-1 expression analyzed by flow cytometry for cells stained with an anti-NY-ESO-1 antibody with mean fluorescent intensity (MFI) depicted. (B) m1G4 $CD8^+$ T cells specifically recognize NY-ESO-1 expression by 1F4 cells as seen by IFNγ production that is HLA-A2 mediated with increasing concentration of blocking antibody (BB7.2) decreasing IFNγ production. Mean with SD error bars of triplicate assays are shown. (C) 1G4 mice (n=5) were injected subcutaneously with 10^6 syngeneic tumor cells, 1F4 (MCA induced tumor from HHD mice). Growth of HHD tumor line expressing human NY-ESO-1 (1F4, purple line), and parental tumor line (B5, blue line) were followed by caliper measurements of tumor size for 5 weeks. Error bars represent SEM.

Table S1. PCR Primers and Conditions used for A2Eso1G4-I Genotyping

PCR For	Primer Name	Primer Sequence 5' -> 3'	PCR Condition
**α TCR**	PCRalphaF	ATGCTGACTGCCAGCCTGTTGAGG	94° 1min 37x(94° 30sec, 57° 40sec, 68° 1min), 70° 10min
	MalphaR	CACGGATGAACAATAAGGC	
**β TCR**	PCRbetaF	ATGGGCTTCAGGCTCCTCTGCTG	See α
	MbetaR	CGGTCAGCCTAGAGCCTTCTCC	
**Mouse Actin**	mActinF	CTGAAGTACCCCATTGAACATGGC	See α
	mActinR	CAGAGCAGTAATCTCCTTCTGCAT	
**Mouse B_2m**	mB2mF	CACGCCACCCACCGGAGAATG	See α
	mB2mR	GATGCTGATCACATGTCTCG	
**Db**	DbForward4	AGTGGTGCTGCAGAGCATTACAAG	94° 1min 37x(94° 30sec, 55° 40sec, 68° 4min), 70° 10min
	DbExon5	AACGATCACCATGTAAGAGTCAGT	
**A2.1 Monochain Presence (het/homo)**	Chr10F1	GTTTCCTGCAGATGCATTATAGAATCAGTTTCACAG	94° 1min 7x(94° 25sec, 60° 30sec, 68° 5min) then 35x(94° 25sec, 50° 30sec, 68° 5min), 68° 10min
	A274GSP1	GACCCACATGAGGTATTAAAAGACAACTGCC	
**A2.1 Monochain Homozygous**	Chr10F1	GTTTCCTGCAGATGCATTATAGAATCAGTTTCACAG	
	Chr10R1	GGACTCAGGTGAGATCCCATTGTTTGTATTG	

Table S2. PCR Primers used for Generation of Full-length NY-ESO-1 Recombinant Vaccinia Virus Encoding SLLMWITQC (9C), SLLMWITQV (9V), and SIINFEKL epitopes.

PCR For	Primer Name	Primer Sequence 5' -> 3'
**Full-length NY-ESO-1 C165V**	P1	GTTGATGTGGATCACGCAGGTCTTTCTGCCCGTGTTTTTG
	P2	CAAAAACACGGGCAGAAAGACCTGCGTGATCCACATCAAC
**Step 1**	FusionmCherry-F	GACGGATCCCGTCGACTCGAGACGCGTATGGTGAGCAAGGGCGAG
	mCherry 3'-SspBI-Ub-Infus2	AGATCTGctTGTACAgctcgtccatgcc
**Step 2**	FusionmUb-F	TGTACAAGcagatcttcgtgaagacgttaac
	Ub 5'-SspBI-mChe-Infus2	GCCTGCATaccacctcttagtcttaagac
**Step 3**	FusionNYESO-F	AggtggtATGCAGGCCGAAGGCCG
	FusionNYESOPSC11R	CAATTCCCAGCGGCCGCTACTTGTCATCGTCGTCCTTG
